# Quantifying and Disclosing the Environmental Footprint of AI in Research: Life Cycle–Informed Framework and Open-Access Calculator Development Study

**DOI:** 10.2196/90770

**Published:** 2026-08-18

**Authors:** Kaveh Mozafari, Yuanchao Ma, Mohsen Amoei, Bertrand Lebouche, Esli Osmanlliu, Dan Poenaru

**Affiliations:** 1 Department of Surgical & Interventional Sciences, McGill University, Montreal General Hospital 1650 Cedar Avenue, T5-110, Montreal, QC, H3G 1A4, Canada, 1 (514) 396-2190; 2 McGill University Health Centre Research Institute, Montreal, QC, Canada; 3 Department of Pediatrics, McGill University, Montreal, QC, Canada; 4 Department of Family Medicine, Faculty of Medicine and Health Sciences, McGill University, Montreal, QC, Canada; 5 Department of Pediatrics, Division of Pediatric Emergency Medicine, Montreal Children's Hospital, Montreal, QC, Canada

**Keywords:** AI, environmental impact, carbon footprint, life cycle assessment, energy consumption, water consumption, sustainable AI, data centers, AI transparency, environmental reporting

## Abstract

**Background:**

AI research increasingly depends on energy-intensive computation, yet energy use, greenhouse gas emissions, hardware life cycle burdens, and water consumption are rarely reported in a standardized way. This lack of reproducible environmental accounting limits comparisons across studies and obscures the trade-offs among model performance, infrastructure choices, carbon intensity, and cooling water demand.

**Objective:**

This study aimed to develop and describe an open-access, life cycle–informed AI Environmental Footprint Calculator and propose a minimum reporting dataset for transparent environmental disclosure in AI research.

**Methods:**

We developed a browser-based calculator that combines operational energy, regional grid carbon intensity, power usage effectiveness (PUE), water usage effectiveness, hardware embodied emissions, and workload-specific metrics for training and inference. The framework was evaluated in 3 representative scenarios: a single–graphics processing unit laboratory fine-tuning task, a midsized academic cluster workload, and a large-scale industrial training cycle. Outputs were compared with those of established tools to identify how boundary choices and parameter assumptions affect emission estimates.

**Results:**

Across the scenarios, the inclusion of PUE and hardware life cycle allocation increased reported emissions compared with operational-only estimates. In the small laboratory scenario, optimization reduced total emissions from approximately 0.07 kg carbon dioxide equivalents (CO_2_e) to 0.05 kg CO_2_e and improved the proposed label from C to B. In the midsized cluster scenario, carbon-aware scheduling reduced emissions from approximately 665 kg CO_2_e/month to 450 kg CO_2_e/month—a 32% reduction. In the large-scale scenario, shifting to renewable-backed, lower-PUE infrastructure reduced operational emissions by approximately 79% while increasing the importance of water-carbon trade-off reporting.

**Conclusions:**

The calculator provides a practical and transparent method for reporting AI environmental footprints using auditable parameters and publication-ready outputs. Routine disclosure of energy use, emissions, water use, hardware assumptions, and regional context can improve reproducibility and support more equitable and sustainable AI research evaluation.

## Introduction

### Study Motivation

AI has become a defining technology of modern science, but its rapid expansion has created a measurable environmental burden through electricity consumption, hardware manufacturing, cooling infrastructure, and water use. Data centers already account for a substantial and growing share of global electricity demand, and recent analyses indicate that AI-oriented infrastructure can intensify local grid demand, operational carbon emissions, and water withdrawals when high-density computing is paired with evaporative or liquid cooling [1-10]. Despite this growth, most AI manuscripts still report computational costs only as graphics processing unit (GPU) hours, floating-point operations (FLOPs), or model size, without translating these quantities into energy, emissions, hardware life cycle burdens, or water use. This prevents meaningful comparison across studies, particularly when the same workload may have very different impacts depending on the region, time of execution, power usage effectiveness (PUE), water usage effectiveness (WUE), and hardware amortization assumptions. The purpose of this study was, therefore, to develop and describe a transparent, open-access AI Environmental Footprint Calculator that integrates operational energy, life cycle hardware emissions, cooling overhead, regional grid intensity, water use, and workload-level metrics into a reproducible reporting framework for AI research.

### Theoretical Background and Literature Review

In response to the growing awareness of AI as a high-energy technology, multiple tools have emerged to quantify the environmental footprint of machine learning (ML) workloads. These frameworks aim to translate computational activity, primarily central processing unit and GPU utilization, into measurable ecological indicators, such as electricity use (kWh) and carbon dioxide equivalent (CO_2_e) emissions, and have become critical first steps toward standardized sustainability reporting in AI research and engineering.

The models described above have been instrumental in encouraging reproducibility and environmental awareness across diverse laboratories. They enable the quantification of sustainability even in institutions without direct metering infrastructure, providing low-barrier entry points for academic users. Their underlying methodology, however, remains grounded in operational energy measurement, typically runtime power × grid intensity, without integrating a life cycle assessment (LCA) of hardware manufacturing, infrastructure, and cooling systems. Recent studies have shown that these unaccounted phases can constitute a large share of the total environmental impact, particularly for large-scale model training and inference [1-6,9].

While these existing tools form the methodological foundation for transparent reporting, their variability in scope, required inputs, and regional assumptions limits comparability across studies. The growing complexity of AI infrastructure calls for next-generation frameworks that incorporate cradle-to-grave accounting, inference energy, and real-time regional grid data, thereby bridging operational measurements with holistic life cycle sustainability analyses [1-9].

### Gaps in Existing Models and the Need for an Accessible Life-Cycle Approach

Recent data-center life cycle studies strengthen the rationale for this broader boundary. The critical analysis by Bux et al [10] of the global warming potential of data centers emphasizes that digital services should be assessed using life cycle thinking rather than only operational electricity because infrastructure, cooling systems, regional grid mix, equipment lifetime, and utilization all shift the resulting footprint. Studies of data-center water and electricity footprints further show that decarbonization strategies may shift impacts between categories: lower-carbon electricity or liquid-cooled facilities can reduce CO_2_e while increasing water demand or local resource pressure [4,7,10,11]. These findings support a calculator design that reports both carbon and water indicators and makes all parameter sources explicit.

Existing carbon-tracking tools, such as those described above, represent essential first steps toward accountability but face several technical and practical limitations that limit their widespread scientific adoption.

First, existing models often lack granularity and completeness: most estimate operational energy during model training by multiplying the average device power by runtime and applying a regional emission factor (EF). This approach can omit hardware manufacturing and end-of-life burdens, cooling energy, and water consumption associated with data-center operation [1-7,10,11].

Second, current models lack sufficient temporal and geographic resolution. Current calculators typically use country-level or annualized carbon-intensity factors, ignoring hourly grid fluctuations and renewable intermittency [7]. Intermittency refers to fluctuations in the supply of renewable energy (eg, solar and wind) over time, which affect grid carbon intensity and the accuracy of emission estimates. This coarse approach can misrepresent actual emissions by factors of 2 to 3, depending on when and where the computation occurs [4,7]. None of the popular frameworks currently integrate real-time grid data or regional proportions of PUE and WUE, despite evidence from Alissa et al [4] and the International Energy Agency (IEA) [7] that such variables significantly alter life-cycle outcomes.

Third, current models offer limited accessibility for nonexperts; most tools require command-line installation, API keys, or specialized logging frameworks, posing challenges for researchers in the health and social sciences, as well as in small academic laboratories. These users often lack permission to install monitoring agents on shared clusters, leaving them dependent on rough manual estimates [2,5,8]. The absence of intuitive interfaces and standardized reporting templates also undermines consistency across publications.

There is, therefore, a clear need for a more accessible, holistic, and precise framework that combines fine-grained life cycle accounting (hardware, operational, and cooling phases) with user-friendly automation. Such a system should integrate seamlessly into existing research workflows, offer meaningful uncertainty ranges, and automatically produce publication-ready environmental reports. This combination of scientific rigor and usability would bridge the gap between advanced sustainability analytics and everyday research practice, supporting broader transparency and reproducibility in AI [1-9].

## Methods

### Calculator Framework and Core Features

The proposed AI Environmental Footprint Calculator [12] (REF-CALC) expands on prior carbon-tracking tools by integrating fine-grained LCA, real-time energy data, and multidimensional environmental indicators within a single platform. Its design philosophy is grounded in the 2025 recommendations for cradle-to-grave transparency in AI systems [1-6,9].

The calculator incorporates the following methodological features:

- Comprehensive boundary coverage: Unlike most operational-only models, the calculator quantifies 4 emission components:
  - Operational energy (runtime GPU and central processing unit power × regional grid intensity)
  - Hardware manufacturing and end-of-life burdens are assessed using embodied carbon factors derived from Schneider et al [1] and Falk et al [6]
  - Cooling and infrastructure overheads are accounted for through dynamic PUE and optional WUE parameters [4,7]
  - Inference energy per output unit is expressed as CO_2_e/1000 tokens or per inference request [3]

- Finer temporal and regional granularity: The model retrieves hourly grid-carbon-intensity data from public APIs (eg, Electricity Maps and IEA datasets) and automatically adjusts calculations based on the user’s selected location, enabling realistic estimates for carbon-aware scheduling scenarios [7].
- Transparent equations and open documentation: All assumptions, conversion factors, emission coefficients, and device lifetimes are visible within the interface. Each calculation is traceable and auditable, aligning with the reproducibility standards advocated by Morrison et al [2] and the Federation of American Scientists (FAS) [8].
- Multi-indicator labeling: In addition to total CO_2_e, the system outputs energy (kWh), water (L), and renewable-energy share, generating a standardized AI environmental label rated from A (very efficient) to E (inefficient). This label mirrors established consumer energy-rating systems and embodies the “sustainable scaling” principles described by Desroches et al [5]. Displaying the label alongside model cards or dataset statements would make environmental performance an explicit part of model evaluation criteria. The A-E grade is assigned by positioning a workload’s carbon intensity, expressed as CO_2_e per 10⁹ FLOPs for training or per 1000 tokens for inference, within a reference distribution constructed from a curated set of published AI workloads and standardized benchmark configurations reported in the recent literature. This reference group includes representative training and inference workloads spanning small academic experiments, midscale research clusters, and large-scale industrial models, using harmonized assumptions for hardware type, utilization, and grid-carbon intensity. In the current implementation, grade A denotes the top 15% of the most carbon-efficient workloads within this reference distribution, grade B the next 20%, grade C the middle 30%, grade D the following 20%, and grade E the bottom 15%. These percentile-based thresholds provide intuitive interpretability while accommodating ongoing improvements in model architecture, hardware efficiency, and energy infrastructure.

Collectively, these features form the core computational framework of the AI Environmental Footprint Calculator. To provide a consolidated view of the 4 components described above, Table 1 summarizes the inputs and computational pathways of the AI Environmental Footprint Calculator. As detailed in the *Calculator Framework and Core Features* section, the calculator integrates hardware life cycle emissions, operational energy use, cooling overhead, and inference-level granularity into a unified estimation pipeline.

These components collectively generate the environmental indicators (kWh, CO_2_e, water use, and A-E label) applied in the validation scenarios presented in the *Results* section.

**Table 1.** Components of the AI Environmental Footprint Calculator and corresponding outputs.

Component	Description	Formula and inputs	Contribution to final output
1. Hardware life cycle assessment	Embodied emissions from manufacturing, transportation, and end-of-life disposal of hardware	Embodied CO_2_e^a^ (kg) per device, amortized over lifetime (h)	Adds embodied CO_2_e component
2. Operational energy	Energy consumed during training or inference	Device power (kW) × runtime (h)	Produces kWh, contributes to CO_2_e, and water (L)
3. Cooling and infrastructure overhead	Data-center overhead energy and cooling water	PUE^b^ × IT energy; WUE^c^ × IT energy	Contributes to additional CO_2_e and water (L)
4. Inference-specific granularity	Per 1000-token or per-request footprint	CO_2_e/1000 tokens from operational+overhead components	Produces inference-level CO_2_e and A-E classification

a CO_2_e: carbon dioxide equivalent.

b PUE: power usage effectiveness.

c WUE: water usage effectiveness.

### Integration With Research Workflows

The calculator is intentionally lightweight and designed to integrate seamlessly into everyday ML workflows without imposing additional software dependencies. Because the tool runs entirely on the client side in a web browser, it requires no installation, can be used on any operating system, and does not access or transmit user data. Researchers can directly input model characteristics, hardware configurations, and workload parameters—or retrieve live hourly grid-carbon intensity values via the integrated Electricity Maps API—to generate reproducible environmental estimates aligned with emerging reporting recommendations for transparency and life cycle completeness [2,7].

All equations, assumptions, emission coefficients, and device lifetimes are fully exposed within the interface, supporting auditable and traceable reporting, consistent with the reproducibility standards emphasized by Morrison et al [2] and the FAS [8]. The calculator also provides inference-specific metrics (eg, CO_2_e per token and per 1000 tokens), automated PUE and WUE adjustments, and intuitive household-energy equivalences to facilitate communication across technical and nontechnical audiences.

By emphasizing accessibility, transparency, and zero-setup use, the tool embeds environmental accounting directly into the researcher’s workflow and lowers the barrier to routine sustainability reporting in ML projects.

### Minimum Reproducible Dataset and Parameter Sources

To address reviewer concerns about reproducibility, the revised framework specifies how each minimum reporting parameter should be measured or documented. Hardware should be reported as the accelerator model, count, memory class when relevant, and hardware life cycle reference source or version, such as a manufacturer disclosure, ecoinvent or Boavizta-style factor, or peer-reviewed LCA. Energy should be reported as measured kWh when direct metering is available, or estimated from device power, utilization, and runtime when metering is unavailable. PUE and WUE should be taken from the facility operator when possible; otherwise, authors should state the assumed value and source. Grid intensity should include the region, date or time window, and whether an hourly API value or annual average was used. Workload information should include total tokens, images, FLOPs, runtime, batch size, or sequence length when applicable, and whether inference or training is being assessed. This structure creates a unified basis for reporting while remaining usable by laboratories without direct metering infrastructure.

### Accessibility and Ease of Use

The interface prioritizes inclusivity across expertise levels:

- For nonexperts and small laboratories: The web version offers a minimal-input mode that requires only the GPU type, runtime hours, and location to produce immediate results with uncertainty ranges.
- For advanced users: An “expert view” unlocks adjustable LCA parameters, grid profiles, and batch-processing capabilities. To enhance interpretability, the calculator not only presents numerical results (kWh and CO_2_e) but also visualizes them through real-world household equivalences, allowing users to relate computational energy use to familiar activities such as lighting, heating, or appliance operation.
- Visualization: Interactive graphs display emissions by source (operations, hardware, and cooling) and over time, enhancing interpretability for grant proposals and sustainability reports.
- No installation barriers: Because calculations run entirely in the browser or via an HTTPS API, the tool functions on institutional machines without administrative privileges, a key limitation noted for prior models [13-15].

By promoting scientific rigor and usability, the calculator enables researchers at any scale, from individual graduate projects to industrial LLM training, to quantify and report their environmental impact with transparency and precision.

### Ethical Considerations

Environmental transparency must coexist with equitable access to computing. The tool, therefore, encourages low-carbon scheduling for publicly funded and academic research while discouraging “grid shopping” practices that merely relocate emissions geographically. This approach supports fair access to clean computing and aligns with the broader goals of responsible AI development.

## Results

### Illustrative Scenarios

To demonstrate the functionality and practical relevance of the proposed calculator, 3 representative scenarios were modeled. Each example corresponds to a different research scale, from an individual small-laboratory training run to a large-scale generative AI deployment, and illustrates how the environmental insights produced by the calculator can guide optimization and decision-making. Table 2 illustrates how the 3 example workloads presented in the *Results* section translate into household electricity use, using common appliances as reference points. For context, all 3 examples below report the corresponding A-E environmental label, where A indicates high efficiency relative to benchmarked workloads and E represents comparatively inefficient processes (see *Calculator Framework and Core Features* section).

**Table 2.** Comparison of representative AI workloads with everyday household electricity use^a^.

Household device or activity	Small laboratory (5 kWh)	Midsized cluster (1900 kWh)	Large-scale industrial training cycle (68,000 kWh)
Energy use (kWh)	5	1900	68,000
60 W light bulb	≈83 h (3.5 d)	≈31,667 h (3.6 y)	≈1.13 million h (129 y)
Laptop (50 W)	≈100 h (4 d)	≈38,000 h (4.3 y)	≈1.36 million h (155 y)
Space heater (1.5 kW)	≈3.3 h	≈1267 h (53 d)	≈45,333 h (5.2 y)
Clothes dryer (2 kW)	≈2.5 h (≈1 load)	≈950 h (≈380 loads)	≈34,000 h (≈13,600 loads)
Refrigerator (150 W)	≈33 h (1.4 d)	≈12,667 h (1.4 y)	≈453,000 h (≈52 y)
Electric vehicle charging (7 kW)	≈0.7 h (~7 km range)	≈271 h (~2700 km)	≈9714 h (~97,000 km)
Average Canadian home electricity (750 kWh/mo)	≈0.007 mo (~5 h)	≈2.5 mo	≈90 mo (~7.5 y)

a Energy consumption from the 3 example scenarios (5 kWh, 1900 kWh, and 68,000 kWh) is expressed as equivalent operation time or quantity for typical household appliances. Appliance power ratings are based on average Canadian household data [7].

### Small Laboratory Fine-Tuning Scenario

**Figure 1.**
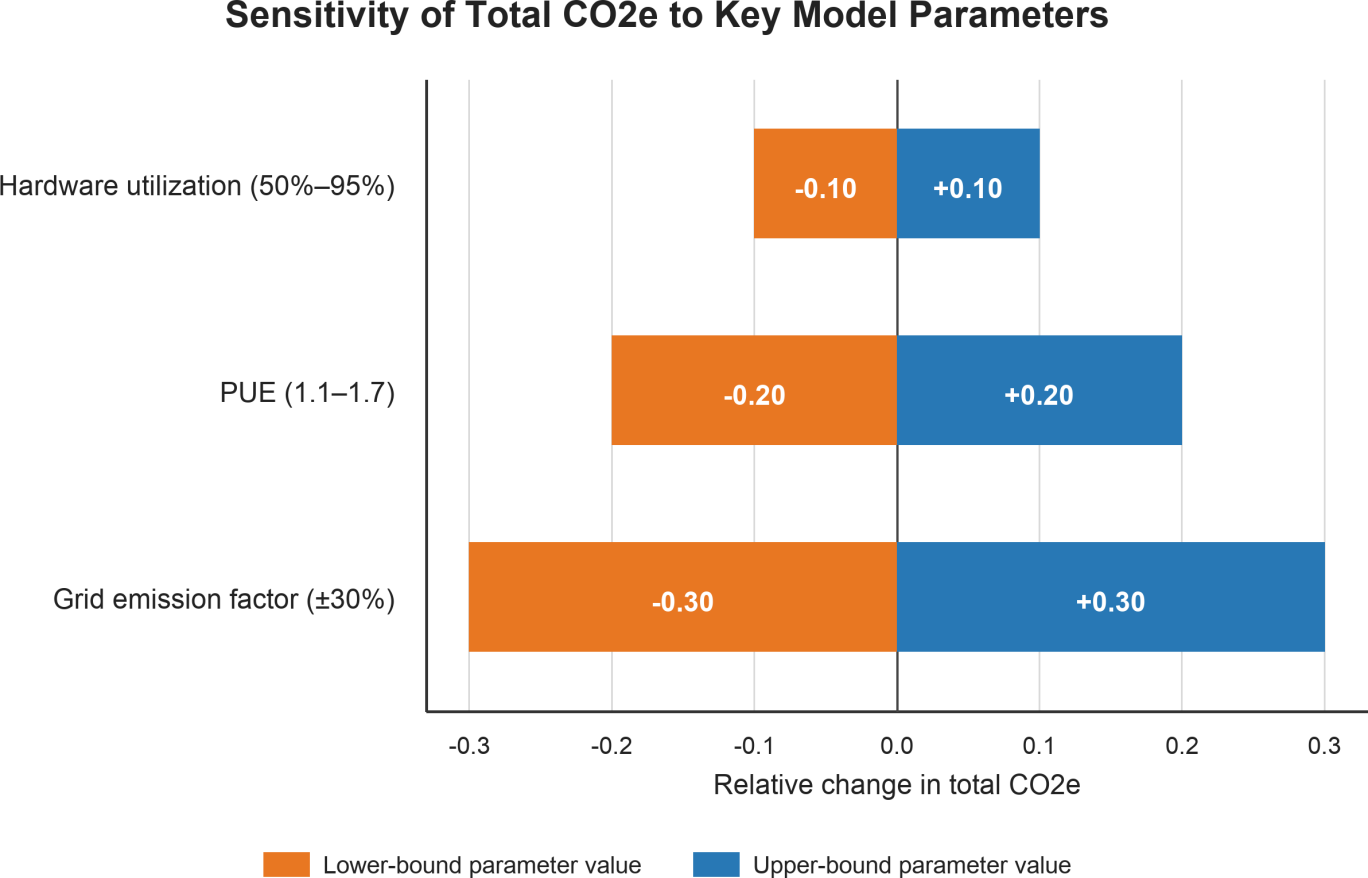
Sensitivity of total carbon dioxide equivalent (CO_2_e) estimates to key model parameters. Horizontal bars show the relative change in total CO_2_e when grid emission factor varies by ±30%, power usage effectiveness (PUE) varies from 1.1 to 1.7, and hardware utilization varies from 50% to 95%. Orange denotes the lower-bound parameter value; blue denotes the upper-bound parameter value. Values shown within the bars are relative changes from the baseline.

A small academic group fine-tuned a pretrained language model (7B parameters) on 1 A100 GPU (Nvidia) for 12 hours in Montréal, where the grid mix is ~95% renewable (Figure 1). The following example illustrates the estimated carbon footprint before and after optimization:

- Initial configuration: Mixed-precision disabled; inefficient data loading resulted in an average draw of ~420 W.
- Baseline result:
  - Operational energy ≈ 5.0 kWh → 0.05 kg CO_2_e (@10 g CO_2_e/kWh)
  - Embodied (amortized) ≈ 0.02 kg CO_2_e [1,6]
  - Total ≈ 0.07 kg CO_2_e → Label “C”
- After optimization: Mixed-precision (FP16) enabled and loaders parallelized, reducing wall time by 35% and average power consumption by 10%.
  - Operational energy ≈ 3.0 kWh; total ≈ 0.05 kg CO_2_e → Label “B”

This case highlights how simple software optimizations can yield material carbon reductions, even where grid emissions are low. The calculator’s disaggregation of operational versus embodied impacts clarifies that the hardware footprint remains dominant when renewable energy is available [1,3,6].

### Midsized Research Cluster Scenario

A university research group operates an 8-GPU node (A100 80 GB) shared among several projects. Using batch import mode, the team uploaded a month’s worth of Slurm Workload Manager logs (SchedMD; ≈1400 GPU-h total).

- Baseline scenario: Continuous queuing produced 1900 kWh total consumption with a regional grid intensity of 0.25 kg CO_2_e/kWh; PUE = 1.4 → CO_2_e ≈ 665 kg/month.
- After implementing carbon-aware scheduling:
  - Jobs dispatched preferentially during low-intensity hours reduced the average EF to 0.17 kg CO_2_e/kWh [7].
  - Operational energy remained unchanged, but total emissions fell to 450 kg CO_2_e (−32%); water consumption also dropped by ≈10% due to cooler nighttime operation [4,7].

The calculator’s hourly-resolved grid factors and visualization dashboard highlight the benefits of time-shifting workloads and document month-to-month trends for sustainability reporting [2,5,7]. Following this optimization, the label improved from C to B.

### Large-Scale Training and Facility Scenario

A commercial consortium trained a 70-B-parameter generative model using 512 A100 GPUs for 24 days (~300,000 GPU-h).

The calculator is integrated:

Assuming a mean accelerator power-draw fraction of 39.4% relative to rated power, estimated IT energy was 512 x 0.45 kW x 0.394 x 24 days x 24 h/day = approximately 52,300 kWh. Applying a PUE of 1.3 resulted in approximately 68,000 kWh of facility energy.

- Grid EF: 0.38 kg CO_2_e/kWh → 25.8 t CO_2_e.
- Embodied hardware (amortized): 0.09 t CO_2_e per GPU × (24 d/3 y ≈ 0.022) → 1.0 t CO_2_e.
- Total: ~26.8 t CO_2_e.

The calculator presents both CO_2_e-WUE trade-offs and per-token metrics:


$$E_{per\;token}=\frac{E_{site}}{N_{tokens}},\;CO_{2}\;e_{per\;token}=E_{per\;token}\times {EF}_{grid}$$

This yielded approximately 0.45 g CO2e per 1,000 tokens before optimization and 0.11 g CO2e per 1,000 tokens after optimization. The resulting label improved from D to B.

This scenario illustrates the calculator’s ability to conduct cradle-to-grave assessments, compare facility scenarios, and quantify trade-offs among carbon, water, and efficiency [1,4-7].

To better contextualize the scale of these computations, the calculator translates each example’s total energy expenditure into familiar household electricity uses. Table 2 illustrates how the same energy values correspond to the operation of standard appliances, ranging from lighting and heating to refrigeration and laundry. These equivalences help users intuitively grasp the physical magnitude of AI workloads by relating them to everyday activities and durations.

Complementing the 3 validation scenarios, we also conducted a sensitivity analysis of the model’s most influential parameters, including grid EF, PUE, WUE, GPU utilization, and hardware lifetime (Multimedia Appendix 1). This assessment confirmed that the calculator responds consistently to realistic variations in parameters and that grid intensity and PUE dominate overall uncertainty. Incorporating both empirical scenarios and parameter-level robustness checks strengthens the validity of the proposed framework.

These comparisons make the calculator’s results more relatable for nonspecialists and demonstrate the societal scale of energy demand across different levels of AI development.

Across different application scales, the calculator consistently produced interpretable metrics that guided meaningful operational improvements. In smaller projects, the most significant reductions came from algorithmic efficiency improvements, with optimized code and precision settings directly reducing energy consumption. At the medium scale, temporal grid variability and carbon-aware scheduling delivered the most significant relative benefits, demonstrating that aligning computation with lower-emission hours can meaningfully reduce footprints. At the largest, hyperscale level, infrastructure design and energy-source selection, such as the use of renewable-backed power or efficient cooling systems, became the dominant factors determining total emissions. Collectively, these findings validate the calculator’s adaptability and show that integrating life-cycle data, temporal resolution, and a user-friendly interface can enable practical decarbonization strategies across diverse research environments [1-9].

### Comparison With Existing Tools

To quantitatively validate the proposed framework, we compared its outputs with CodeCarbon and the Green Algorithms Calculator across the 3 validation scenarios (Table 3). The differences between the tools were not interpreted as errors; rather, they reflect distinct methodological boundaries. CodeCarbon primarily estimates operational emissions from device power, runtime, and grid intensity, so it yields lower values when cooling and hardware life cycle burdens are excluded. Green Algorithms includes a broader data-center energy adjustment through PUE but generally does not allocate accelerator manufacturing impacts at the workload level. The proposed calculator reports higher totals when PUE, WUE, and embodied hardware allocation are included because it uses a life-cycle boundary that includes facility overhead and accelerator manufacturing. Therefore, the variation in Table 3 can be attributed mainly to 4 variables: whether cooling overhead is included, whether hardware life cycle emissions are amortized to the workload, whether grid factors are static or time-resolved, and whether inference or per-unit workload metrics are reported. Making these boundary choices visible is a central contribution of the calculator because it allows readers to understand why 2 tools can produce different emissions estimates for the same AI workload.

**Table 3.** Quantitative comparison of environmental impact estimates across tools for the 3 validation scenarios^a^.

Scenario and tool	Energy Estimate (kWh)	Cooling included (PUE^b^)	Hardware LCA^c^ included	Total CO_2_e^d^
Small laboratory (1 × A100, 12 h, Montreal)				
CodeCarbon	5.0	No	No	0.05 kg
Green Algorithms	5.0	Yes	No	0.06 kg
Proposed calculator	5.0	Yes	Yes	0.07 kg
Midsized cluster (8 × A100, monthly)				
CodeCarbon	1900	No	No	475 kg
Green Algorithms	1900	Yes	No	520 kg
Proposed calculator	1900	Yes	Yes	665 kg
Large-scale training (512 × A100, 24 d)				
CodeCarbon	52,000	No	No	19.8 t
Green Algorithms	55,000	Yes	No	22.5 t
Proposed calculator	68,000	Yes	Yes	26.8 t

a Energy estimates are not uniform outputs across tools. Values reflect each tool’s power, utilization, and system-boundary assumptions. CodeCarbon primarily reports estimated IT energy, whereas facility overhead may be incorporated through PUE by Green Algorithms and the proposed calculator. In the proposed calculator’s large-scale scenario, estimated IT energy was approximately 52,300 kWh and facility energy after application of PUE = 1.3 was approximately 68,000 kWh. Differences in total CO2e also reflect grid-emission factors, cooling treatment, and whether amortized hardware life-cycle emissions are included.

b PUE: power usage effectiveness.

c LCA: life cycle assessment.

d CO_2_e: carbon dioxide equivalent.

## Discussion

### Principal Findings

This study introduced a life cycle–informed calculator and reporting framework for AI environmental disclosure. The main finding is that AI footprint estimates change substantially when the reporting boundary expands from operational electricity alone to include PUE, WUE, hardware life cycle allocation, and regional grid intensity. Across the 3 scenarios, the calculator produced interpretable estimates that showed how software optimization, carbon-aware scheduling, and facility selection can reduce emissions while also revealing cases where carbon reductions may increase water demand. These findings support the need for transparent, parameter-level reporting rather than a single, unqualified emissions value.

Environmental transparency should, therefore, be considered a fundamental element of research integrity. Just as ethical statements and data availability sections have become standard components of scientific reporting, so too should *environmental impact disclosures* become routine in computational publications. The inclusion of such information allows peers, reviewers, and the broader public to evaluate research not only by its accuracy and novelty but also by its ecological responsibility [2,5,8].

To operationalize this vision, we propose that AI journals, conferences, and institutional repositories adopt a dedicated “Environmental Impact Statement” or “Carbon Footprint Reporting” section for all computational manuscripts. The AI Environmental Footprint Calculator [12] enables researchers to automatically generate this information, reporting total energy use (kWh), emissions (CO_2_e), grid region, and key infrastructure parameters in a consistent, auditable format [2,8].

Integrating such disclosures will not only improve scientific reproducibility but also catalyze the cultural shift toward sustainable computing. As the 2025 *Energy and AI* report by the IEA notes, transparency is the essential first step toward measurable emissions reduction and informed infrastructure planning [7]. With clear reporting tools and standardized metrics in place, the research community can collectively work toward a lower-carbon future for computational science.

To translate these principles into practical action, the following subsection outlines specific policy and practice recommendations for institutions, publishers, and researchers.

### Policy and Practice Framework

Building on the principles of transparency and accountability outlined above, this subsection translates these commitments into actionable recommendations for researchers, institutions, and publishers.

- Requesting transparent and standardized reporting in publications: Every AI research article should include a concise, standardized environmental reporting subsection that details emissions from training, inference, hardware production, and facility operations. This subsection can be automatically generated through tools such as the AI Environmental Footprint Calculator [12], which outputs structured text and bibliographic references suitable for insertion into the methods section [2,8]. Including such a disclosure normalizes sustainability reporting and facilitates direct comparison across studies.
- Adopting a community-wide environmental label: To complement numerical disclosure, journals and conferences should encourage the use of an AI Environmental Label, a simple A-E grade derived from total CO_2_e per 10⁹ FLOPs (training) and per 1000 tokens (inference).
- Minimum dataset for reproducibility: For each computational experiment, authors should report at least the following parameters (adhering to this dataset ensures reproducibility and enables downstream meta-analyses of AI’s environmental trends):
  - Hardware: GPU or Tensor Processing Unit (Google) model and count, memory size, and hardware LCA reference source or version [1,6,9]
  - Energy: Total kWh, measured or estimated PUE, and WUE if available [4,7]
  - Grid: Geographic region and either hourly grid-EFs or an averaged intensity with a date range [7]
  - Workload: Total FLOPs or token count, batch and sequence length, and mean utilization rate [2,8]
  - Inference disclosure: Per-1000-token or per-request footprint using standardized formulas [3]
- Positive-sum efficiency guidance: Environmental transparency should not be viewed as a constraint but as an opportunity for efficiency gains. Algorithmic improvements, such as mixed-precision training, model pruning, or distillation, can simultaneously reduce cost and emissions. Similarly, carbon-aware scheduling and renewable-backed compute supply can minimize CO_2_e without sacrificing performance [2,5,7]. Embedding these practices into research culture will allow sustainable computing to advance in parallel with scientific innovation.

Together, these recommendations provide a practical roadmap for integrating environmental accountability into computationally intensive AI research. By institutionalizing disclosure, labeling, and reproducibility standards, the community can align methodological transparency with planetary sustainability. Importantly, this framework underscores that environmental assessment must become a routine and integral component of evaluating any AI project [1-9].

### Summary of Contributions

This work introduces the AI Environmental Footprint Calculator. This fully transparent, browser-based tool integrates 4 core components of environmental accounting for AI workloads: (1) operational energy use derived from hardware power measurements and utilization, (2) life cycle–based embodied emissions of accelerators, (3) cooling and facility overheads via configurable PUE and WUE parameters, and (4) inference-level granularity expressed as CO_2_e per token and per 1000-token output. The calculator additionally incorporates live grid carbon intensity data from the Electricity Maps API, a standardized A-E environmental efficiency label, and household energy-equivalent comparisons to support interdisciplinary communication. These elements collectively address ongoing calls in the 2025 literature for transparent, reproducible, and accessible AI environmental reporting frameworks [1-9].

### Comparison With Existing Tools and Methodologies

While prior tools, such as CodeCarbon, ML CO_2_ impact, and Green Algorithms, have advanced operational carbon tracking for ML workloads, they differ from the present tool in several respects (Table 4). CodeCarbon emphasizes integration with Python workflows and local logging but provides limited inference-level granularity and does not incorporate hardware-embodied emissions by default. Green Algorithms provides life-cycle components and region-based performance factors but does not expose the underlying equations directly through the interface. The present calculator complements these efforts by prioritizing transparency, allowing users to inspect and modify every assumption, including hardware lifetime, embodied emissions, PUE and WUE factors, and grid intensity, directly in the interface without requiring installation or coding. In addition, the A-E label introduced here offers an interpretable ranking system explicitly tied to CO_2_e per 1000 generated tokens, a level of granularity not commonly supported in existing tools. Together, these features position the calculator as a methodological bridge between simple footprint estimators and complete LCA approaches. This study is methodological in nature and does not involve hypothesis testing or statistical inference using *P* values.

**Table 4.** Comparison of main tools for estimating AI carbon footprints^a^.

Tool or model	Type and platform	Input requirements	Outputs	Training vs inference coverage^b^	Ease of use and target user	Main limitations^c^
CodeCarbon	Python library (open source)	Device type, power draw (auto), run time, region (ISO country code)	CO_2_e (kg), energy (kWh)	Primarily training (can log inference with wrappers)	Moderate ease of use; intended for ML^d^ practitioners and researchers with Python experience. Requires installation and integration into training scripts but provides automated logging once configured.	Uses static grid factors; ignores embodied hardware and cooling effects [13]
ML CO_2_ impact	Web calculator	FLOPs^e^ or GPU^f^ hours, region, PUE^g^	CO_2_e (kg), equivalent cars or flights	Training only	High ease of use; intended for nontechnical researchers and authors. Fully browser-based with manual input fields, requiring no installation or programming knowledge.	Manual inputs only; no live metering; training focus [14]
Carbon tracker	Python and CLI^h^	Power sensor (nvidia-smi), job duration, grid intensity API	Energy (kWh), CO_2_e (kg) over time	Training (predictive during run)	Low-to-moderate ease of use; intended for system administrators and advanced ML users. Requires Linux-based servers, GPU power monitoring, and command-line execution, limiting accessibility for nonexpert users.	Does not track inference or hardware manufacturing [15]
Experiment impact tracker	Python library (open source)	GPU and CPU^i^ utilization, runtime logs	Energy (kWh), CO_2_e (kg)	Training	Low ease of use; intended for advanced research teams with experiment-management infrastructure. Requires detailed runtime logging and integration into experimental pipelines, making setup complex for small laboratories.	Complex setup, local power profiling required [15]
Green Algorithms calculator	Web app+spreadsheet model	Hardware type, runtime, memory use, data-center PUE	Energy (kWh), CO_2_e (kg)	General scientific computing (training and inference both possible)	High ease of use; intended for general scientific researchers, including non-ML users. Graphical web interface with simplified inputs enables use without programming or system-level access.	Based on generic emission factors, no GPU-specific calibration [16]

a Each tool converts compute power and runtime into carbon dioxide equivalent (CO_2_e) using regional grid factors.

b Phases of the machine learning workflow supported by the tool, including training, inference, or both.

c Low granularity, missing embodied impacts, and static grid assumptions.

d ML: machine learning.

e FLOPs: floating-point operations.

f GPU: graphics processing unit.

g PUE: power usage effectiveness.

h CLI: command-line interface.

i CPU: central processing unit.

### Interpretation of Findings and Practical Implications

Across the 3 validation scenarios, the calculator consistently demonstrates how environmental impacts scale nonlinearly with model size, token throughput, and cluster configuration. Even modest inference workloads can accumulate operational emissions when multiplied across large user bases or when operated in carbon-intensive regions. Conversely, workloads executed in low-carbon grids (eg, Quebec) with efficient cooling infrastructures exhibit markedly reduced footprints, underscoring the opportunity for carbon-aware scheduling and region selection. The A-E label further contextualizes these impacts by providing a simple benchmark for comparing workloads across models, research groups, and deployment environments. For nonexpert stakeholders, including clinicians, policymakers, and general audiences, the household-equivalent comparisons offer a relatable way to interpret energy use that can support informed discussions about AI deployment, sustainability trade-offs, and responsible scaling.

### Limitations, Equity, and Scope

Despite its strengths, the calculator has several limitations. First, embodied emissions values for accelerators remain uncertain because public LCAs and manufacturer disclosures vary in scope, allocation methods, and assumed lifetimes. Second, the Electricity Maps and similar APIs provide high-quality data for many regions, but coverage remains incomplete, requiring some users to rely on static averages. Third, training workloads involving distributed optimization, communication overheads, and dynamic scheduling can deviate from simplified FLOP- and runtime-based estimates. Fourth, the calculator does not yet ingest cloud billing records, cluster logs, or Python runtime traces automatically. Finally, environmental labels must be interpreted carefully across regions. Researchers in countries with carbon-intensive grids may receive lower labels for reasons outside their direct control, which could create unfairness if labels were used as punitive evaluation metrics. We therefore recommend that labels be reported with regional context and used to encourage transparent mitigation strategies, not to penalize researchers for infrastructure constraints. Equity-oriented interpretation should distinguish controllable choices, such as model efficiency and scheduling, from structural constraints such as national grid mix and access to low-carbon compute.

### Future Directions

Future development will focus on expanding interoperability with research workflows via optional Python and command-line interface wrappers, batch import of Slurm Workload Manager and Kubernetes logs, and automated integration with ML frameworks such as PyTorch Lightning and Hugging Face Trainer. Extending API support to additional sources (eg, IEA datasets, regional utility providers) will improve the robustness of real-time grid intensity estimation. Enhancing the embodied-emission module with more granular manufacturing and supply-chain data, including memory-specific and interconnect components, would further strengthen life-cycle accuracy. Additionally, aligning the A-E environmental label with wider community standards, or integrating it into a broader disclosure framework, may facilitate adoption in peer-reviewed publications and regulatory contexts. As transparency expectations in AI research continue to increase, lightweight tools such as the one presented here can play a central role in fostering accountable, environmentally aware development and deployment of AI systems.

### Conclusions

This work introduced a comprehensive, life cycle–aware framework for quantifying and reporting the environmental footprint of AI systems. Through the development of the AI Environmental Footprint Calculator, we provided a transparent, user-friendly, and scientifically rigorous tool that integrates hardware life cycle emissions, operational energy, cooling overhead, and inference costs into a single, reproducible assessment model.

Validation across 3 representative scenarios—a small laboratory fine-tuning task, a midsized academic cluster, and a large-scale industrial training cycle—demonstrates the calculator’s flexibility and practical value (Multimedia Appendix 2). These examples confirmed that algorithmic optimization, carbon-aware scheduling, and renewable-backed infrastructure can collectively achieve meaningful emission reductions without constraining research output [2,4,5,7].

The policy recommendations outlined in the *Principal Findings* section establish a clear path forward: integrating standardized environmental reporting into every AI publication, adopting a universal labeling framework, and defining a reproducibility dataset that includes hardware, energy, grid, and workload parameters. Together, these steps can institutionalize environmental accountability in computational research.

Looking ahead, the next phase of this work will involve expanding the open-access hardware LCA registry, linking the calculator to cloud-platform APIs for automated data retrieval, and collaborating with publishers and funding agencies to pilot environmental reporting requirements. Embedding these practices across the research life cycle will ensure that the progress of AI remains aligned with the broader goal of planetary sustainability.

## Supplementary material

10.2196/90770 Multimedia Appendix 1 Methods, uncertainty and sensitivity analysis, and ethics notes.

10.2196/90770 Multimedia Appendix 2 Supplementary uncertainty analysis and methodological details for the AI Environmental Footprint Calculator, including Figures S1 and S2, the amortization policy, water and thermal trade-offs, and the mathematical framework.

## Abbreviations

CO_2_e: carbon dioxide equivalent
EF: emission factor
FAS: Federation of American Scientists
FLOP: floating-point operation
GPU: graphics processing unit
IEA: International Energy Agency
LCA: life cycle assessment
ML: machine learning
PUE: power usage effectiveness
WUE: water usage effectiveness
