# Supplementary material for: Quantifying and Disclosing the Environmental Footprint of AI in Research: Life Cycle–Informed Framework and Open-Access Calculator Development Study
Source: JMIR AI. 2026 Aug 18;5:e90770. doi: 10.2196/90770 (PMC13485112; doi:10.2196/90770)
Supplement: Supplementary file 1 [file ai-v5-e90770-s001.docx]

# Multimedia Appendix 1: Supplementary Methods, Sensitivity Analysis, and Ethics Notes

## Uncertainty and Sensitivity Analysis

To ensure methodological robustness, the calculator includes a built-in module for uncertainty and sensitivity analysis. Users can vary key parameters—grid emission factor (±30%), Power Usage Effectiveness (PUE = 1.1–1.7), and hardware utilization (50–95%)—to visualize their influence on total CO₂e. The resulting sensitivity and uncertainty outputs are shown in Supplementary Figure S1 (tornado plots) and Supplementary Figure S2 (95% confidence interval bands), highlighting which parameters dominate uncertainty across use cases.

| *Parameter* | *Symbol* | *Default Value / Range* | *Units* | *Description / Source* |
| --- | --- | --- | --- | --- |
| *Grid Emission Factor* | EF | ±30 % variability | kg CO₂e / kWh | Carbon intensity of regional grid [1–9] |
| *Power Usage Effectiveness* | PUE | 1.1–1.7 | – | Data-center energy overhead factor [4,7] |
| *Water Usage Effectiveness* | WUE | 0.5–3.0 | L / kWh | Water use per energy unit for cooling [4,7] |
| *GPU Utilization* | U | 50–95 % | % | Average device utilization during operation |
| *Hardware Lifetime* | L | 24 000 h (~3 years) | h | Amortization period for embodied emissions [1,9] |
| *Grid Region / Time* | — | Hourly or average EF | – | Temporal and spatial variation of energy source [7] |

**Table A1.** Key parameters used in the uncertainty and sensitivity analysis of the AI Environmental Footprint Calculator. Default values and ranges are derived from 2025 lifecycle and energy-assessment studies of AI systems [1–9]. Parameters include grid-emission factor (EF), Power Usage Effectiveness (PUE), Water Usage Effectiveness (WUE), GPU utilization, and hardware lifetime used for embodied-emission amortization.
