# Supplementary material for: Quantifying and Disclosing the Environmental Footprint of AI in Research: Life Cycle–Informed Framework and Open-Access Calculator Development Study
Source: JMIR AI. 2026 Aug 18;5:e90770. doi: 10.2196/90770 (PMC13485112; doi:10.2196/90770)
Supplement: Supplementary file 2 [file ai-v5-e90770-s002.docx]

**Multimedia Appendix 2**

*Supplementary uncertainty analysis and methodological details for the AI Environmental Footprint Calculator*

Supplement to “Quantifying and Disclosing the Environmental Footprint of Artificial Intelligence in Research”

**Figure S1.** Sensitivity of total carbon dioxide equivalent (CO2e) estimates to variation in grid emission factor, power usage effectiveness, and hardware utilization. Bars show the relative change in total CO2e across the stated parameter ranges.

# Supplementary Figure S2

**Figure S2.** Uncertainty in total carbon dioxide equivalent (CO₂e) estimates across the small-laboratory, mid-sized-cluster, and large-scale-training scenarios. Points indicate central estimates; vertical bars indicate 95% confidence intervals derived from simultaneous variation in grid emission factor, power usage effectiveness, and hardware utilization.

# Amortization Policy

Embodied manufacturing emissions for hardware are amortized by default over a 3-year lifetime or 24,000 operating hours per accelerator, consistent with Schneider et al [1] and Plociennik et al [9]. Users may override these defaults to reflect refurbishment or second-life scenarios. Both attributional (per-use allocation) and consequential (marginal replacement) perspectives are supported, allowing flexibility in life cycle reporting.

# Water and Thermal Trade-Offs

Because cooling technologies strongly influence both carbon and water footprints, the calculator explicitly models the CO₂e–water usage effectiveness trade-off space. For example, shifting from air cooling to liquid cooling can reduce operational emissions by 30%–70% but can increase water consumption, depending on regional climate conditions [4,7]. Users can explore these trade-offs interactively to guide sustainable facility planning.

# Mathematical Framework

The calculator’s computations follow a transparent, auditable structure:

| **IT energy** | E_IT = P_cluster × t_workload |
| --- | --- |

| **Facility energy** | E_facility = E_IT × PUE |
| --- | --- |

| **Operational emissions** | CO₂e_ops = Σ_t E_facility(t) × EF_grid(t) |
| --- | --- |

| **Embodied emissions** | CO₂e_emb = n_devices × embodied_CO₂e_device × (t_workload / lifetime_hours) |
| --- | --- |

| **Total emissions** | CO₂e_total = CO₂e_ops + CO₂e_emb |
| --- | --- |

| **Water use** | Water_total = E_facility × water_intensity + E_IT × WUE |
| --- | --- |

For inference workloads, per-unit values are calculated by dividing total energy, emissions, or water by the number of tokens, requests, or images.
